# Estimating and validating the structure of feeding behavior networks

**DOI:** 10.1007/s40519-022-01489-1

**Published:** 2022-10-16

**Authors:** Hao Zhang, Xinrui Li, Zhou Lu, Haiyue Zhang, Zhe Yang, Yue Wang, Yuhai Zhang, Xun Jiang, Lei Shang

**Affiliations:** 1 Department of Health Statistics, School of Public Health, Fourth Military Medical University, Xi’an, 710032 China; 2 Department of Health Service, Training Base for Health Service, Fourth Military Medical University, Xi’an, China; 3 Department of Pediatrics, Tangdu Hospital, Fourth Military Medical University, Xi’an, 710038 China; 4 Medical Department, PLA 985th Hospital, Taiyuan, 030001 China

**Keywords:** Network analysis, Feeding behavior, Scale structure, Validity

## Abstract

**Purpose:**

Network analysis has been widely used in psychometrics over the past decade, yet it is unknown that whether this methodology could be applied in the field of child health assessment such as caregivers’ feeding behavior and child eating behavior. Our study leveraged network psychometrics method to estimating and examining the network structure of Chinese Preschoolers’ Caregivers’ Feeding Behavior Scale (CPCFBS), and compared the applicability of network methods in the feeding behavior scale.

**Methods:**

The CPCFBS was previously applied in a sample of 768 preschoolers’ caregivers, used to estimate the structure of feeding behavior networks. Network structure was estimated with Gaussian Graphical Model. Dimensionality was detected using Exploratory Graph Analysis (EGA). The network structural consistency was tested using EGA bootstrap. The network structure was compared with the original structure using model fit indices and reliability.

**Results:**

A seven-dimensional EGA network was explored after rearranging four items and deleting one item with unstable structural consistency. The absolute fit and relative fit of EGA structure were better than the original structure. The EGA structure had nearly same values of the reliability with the original structure.

**Conclusion:**

Our study presented a novel perspective for feeding behavior analytical strategies, and demonstrated that network analysis was applicable and superior in exploring the structure of feeding behavior scales.

**Level of evidence:**

Level V, cross-sectional descriptive study.

**Supplementary Information:**

The online version contains supplementary material available at 10.1007/s40519-022-01489-1.

## Introduction

Childhood obesity has become a serious public health problem in China nowadays [1]. Studies have shown that many metabolic diseases, cardiovascular diseases, and cancers were closely related to obesity in childhood [2]. The age of 3–6 years is the preschool age in China and is also a critical period for obesity prevention [3]. Evidence suggested that caregivers’ feeding behaviors shape children’s eating habits and play an important role in childhood obesity [4, 5]. Caregivers control when, what, and how their children eat and drink, as well as provide the eating environment to set the emotional tone for eating occasions [5]. It is therefore critically important to identify inappropriate caregivers’ feeding behaviors in children’s early stage.

Feeding behavior is a complex system associated with a variety of factors. To accurately assess feeding behavior, several scales were developed and validated based on different race, culture, and eating habits [5–7]. Recently, Chinese Preschoolers’ Caregivers’ Feeding Behavior Scale (CPCFBS) has been developed and evaluated using standard development process [8]. The researchers selected items and extracted dimensions accordingly based on the characteristics of Chinese dietary culture, such as feeding environment of single-child families and caregivers’ excessive concern about children’s weight [8]. As a validated scale, the CPCFBS has been promoted for use in some regions of China.

Scale development is mostly concerned with measurement construction and performance evaluation. Traditional measurement theory [e.g., Classical Test Theory (CTT), Generalizability Theory, and Item Response Theory] consider latent variable models as a standard conceptualization of measurement [9] in which observational variables were seen to be incurred by a common underlying cause [10]. Nevertheless, network analysis elaborates on the occurrence of observational variables as led by their mutual associations and interactions and thus to form an interconnected network [11]. Mutual influence among variables is usually measured by regularized partial correlations [12]. Network analysis has been widely used to explore structures of psychological, biological, and other systems [13] and researchers have demonstrated that network analysis can be used as a new approach to identify the structure of complex systems with interacted elements [11, 14, 15]. Network analysis informs a novel perspective to understand scales [16]; however, it has not been developed in the use of children’s health monitoring.

In this study, we leveraged network psychometrics method to conduct an exploratory study of the CPCFBS by estimating the network structure, using a large-scaled dataset of Chinese caregivers. Based on the network analysis approach and previous research, we expected to provide a novel perspective on the application of network methods, aiming at demonstrating how the network model can be applied to the establishment of dataset and the validation of network structures (e.g., dimensionality, structural consistency) in the feeding behavior scale research areas.

## Methods

### Data

Data were from the CPCFBS research [8] which specifically aimed at urban and rural caregivers whose children were in kindergartens located in the city of Jinan and Xi’an, China. The inclusion criteria for the participants and data collection procedures were presented in detail in the literature [8]. A sample of 768 preschoolers’ caregivers was recruited at baseline. The caregiver was defined as “the primary caregiver cared for the child’s daily living (e.g., diet, sleeping, and activity) at home after school and over the weekend” [8]. All participants completed the CPCFBS in its entirety. Demographic characteristics of the participants are shown in Table S1. Among all participants, 52% were from urban and 48% were from rural area, 76.2% were the children’s parents and 23.8% were the children’s grandparents and others. 53.4% of the children were male and 46.6% were female. The age of children ranged from 3 to 6 years (*M* = 4.9 years old, *SD* = 1.0), of which 31.5% were 3 to 4 years old, 33.5% were 4 to 5 years old, and 35.0% were 5 to 6 years old. The median caregiver education level was senior high school. The median family monthly income was $750-$1500.

### Measures

The CPCFBS developed by Jing Yuan [8] was used in our investigation. The measure was comprised of 35 items and seven dimensions: *Responsibility for feeding*, *Weight concerns*, *Content-restricted feeding*, *Behavior-restricted feeding*, *Encourage healthy eating*, *Forced feeding, *and* Supervise eating*. The items were measured on a 5-point Likert scale, ranging from 1 “never” to 5 “always”. According to the manual, reverse items were negatively scored, and higher scores of each dimension indicated a greater tendency of caregivers to feed their children in this manner.

The CPCFBS was developed according to a strict standard scale development process. CTT statistical method was used to select items. The main techniques used in CTT to assess the data were Principal Component Analysis and factor analysis, which was constructed based on latent variable models and focused on extracting common covariates among variables to generate factors [17].

### Statistical analysis

The statistics analysis was conducted by R 4.0.4, with package of *bootnet* [18], *EGAnet* [19], *qgraph* [20], *lavaan* [21], and *MBESS* [22].

### Network estimation and visualization

A network comprised variables (e.g., CPCFBS items) that were presented by nodes, and edges between nodes represented the associations between variables [23]. For our multivariate normal data, we chose the *qgraph* and *bootnet* package to estimate the CPCFBS network with Gaussian Graphical Model (GGM), in which edges represented partial correlations between nodes [24]. It was critically important that the associations in GGM construction helped us distinguish the risky feeding behavior of preschoolers’ caregivers [23]. As the number of nodes increased, more edges would be estimated. However, since many edges are spurious correlations, the larger number of nodes may lead to model over-fitting and unstable network [23, 25]. The Graphical Least Absolute Shrinkage and Selection Operator (GLASSO) was used to penalize and shrink edge weights, and set edges with small partial correlations to zero to result in a sparse network that reflects only the most accurate edges [26]. We estimated the best-fitting network model using the Extended Bayesian Information Criterion (EBIC) [27, 28], the tuning parameter $\gamma$ of EBIC determines the sparseness of the network. The higher value of $\gamma$(ranges from 0 to 1), the more parsimonious the models would be estimated [12]. The function “EBICglasso” in *bootnet* package was used with the default value of $\gamma$ = 0.5. To visualize the network system, the Fruchterman–Reingold algorithm was used to determine nodes layout [29], which distributed strong connected nodes closer to the center of the network or otherwise decentralized [23].

### Dimension detection

In the network, nodes form clusters (communities, dimensions) according to the strength of the relevance. Recent studies have proved that network was statistically equivalent to traditional latent variable model, yet their mechanisms were different [30]. To accurately estimate the number of dimensions of CPCFBS networks, we employed EGA, which outperformed the traditional factor analysis methods (e.g., exploratory factor analysis, parallel analysis) [31]. EGA applied the default community detection algorithm *walktrap *[32] to investigate the number of communities and automatically classify items to their corresponding community. The algorithm using “random walks” iteratively traversed over neighboring edges, with larger edge weights being more probable paths of travel, a community was then detected by its proportion of densely connected edges to sparsely connected edges [33]. The output of the network detected by EGA was a plot that the nodes of the same cluster were colored separately to give an intuitive visual interpretation [34].

After dimensionality was detected using the *EGAnet* package, we calculated *network loadings* which is roughly equivalent to factor loadings [35]. Network loadings indicate the contribution of each item to more than one dimension (i.e., cross-loading) and the items that are poorly related to any dimensions [35]. Compared to factor loadings, network loadings are evaluated after the number of factors has been extracted from the network’s structure. Nodes are assigned to particular domains via a community detection algorithm rather than the traditional factor analytical standard of their largest loading in the loading matrix [35]. Christensen and Golino [35] recommended effect size guidelines: small (0.15), moderate (0.25), and large (0.35) network loadings.

### Structural consistency

Structural consistency is defined as the extent to which causally coupled nodes cluster a coherent community within a network [36]. We evaluated structural consistency by the parametric bootstrap EGA (*bootEGA*) [33] (2500 bootstrap samples), which estimated a network and generated new replications with the same number of nodes as the original network, and then repeated the step until the desired number of bootstrap samples and the replicated datasets were achieved [37]. We explained structural consistency in two ways: (1) frequencies that the dimensions identified, and (2) frequencies that nodes clustered into their particular dimensions as well as other dimensions in the replicated datasets. The latter approach was called *item stability *[37]. Lower item stability indicated lower structural consistency [38]. A value of 0.75 was set as an acceptable standard for the dimension replication and item stability, advised by Christensen and Golino (2019).

### Model fit

Previous studies suggested that the network model and latent variable model can be implemented to explain the variance–covariance structure of observational curious variables [39]. The two approaches are alternative to each other. To examine the model fit, we compared the original structure, EGA (all) structure, and EGA (del) structure via the *absolute* fit and the *relative* fit [40]. The absolute fit indicates whether the item responses can be properly interpreted by the dimensional structure. The relative fit indicates which dimensional structure is more suitable as opposed to others. We evaluated the absolute fit using the values of the root-mean-square error of approximation (RMSEA) and the comparative fit index (CFI). The values of RMSEA ≤ 0.05 and CFI ≥ 0.97 indicate a good absolute fit, RMSEA ≤ 0.07 indicate an acceptable absolute fit, and CFI between 0.95 and 0.97 is considered acceptable. The lower value of the Akaike Information Criterion (AIC) and the Bayesian Information Criterion (BIC) indicate the better relative fit [39]. The *lavaan* package was used for this analysis.

### Reliability

To date, Cronbach’s alpha coefficient is the most popular approach of reliability. However, researchers suggested a more sensible index of internal consistency: McDonald’s Ω [41]. The McDonald’s Ω outperformed Cronbach’s alpha coefficient in situations such as (A) fewer and more realistic assumptions, and (B) fewer problems associated with inflation and attenuation of internal consistency estimation [22]. We used the R package *MBESS* to investigated the reliability of full subscale of the original CPCFBS structure and EGA structure with the McDonald’s Ω and Cronbach’s alpha coefficient. For all 95% Cis, coefficients were computed across 1000 bootstrap samples. Same as Cronbach’s alpha coefficient, the score of McDonald’s Ω above 0.7 was considered satisfactory for internal consistency [42].

## Results

### Network estimation

**Fig. 1:**
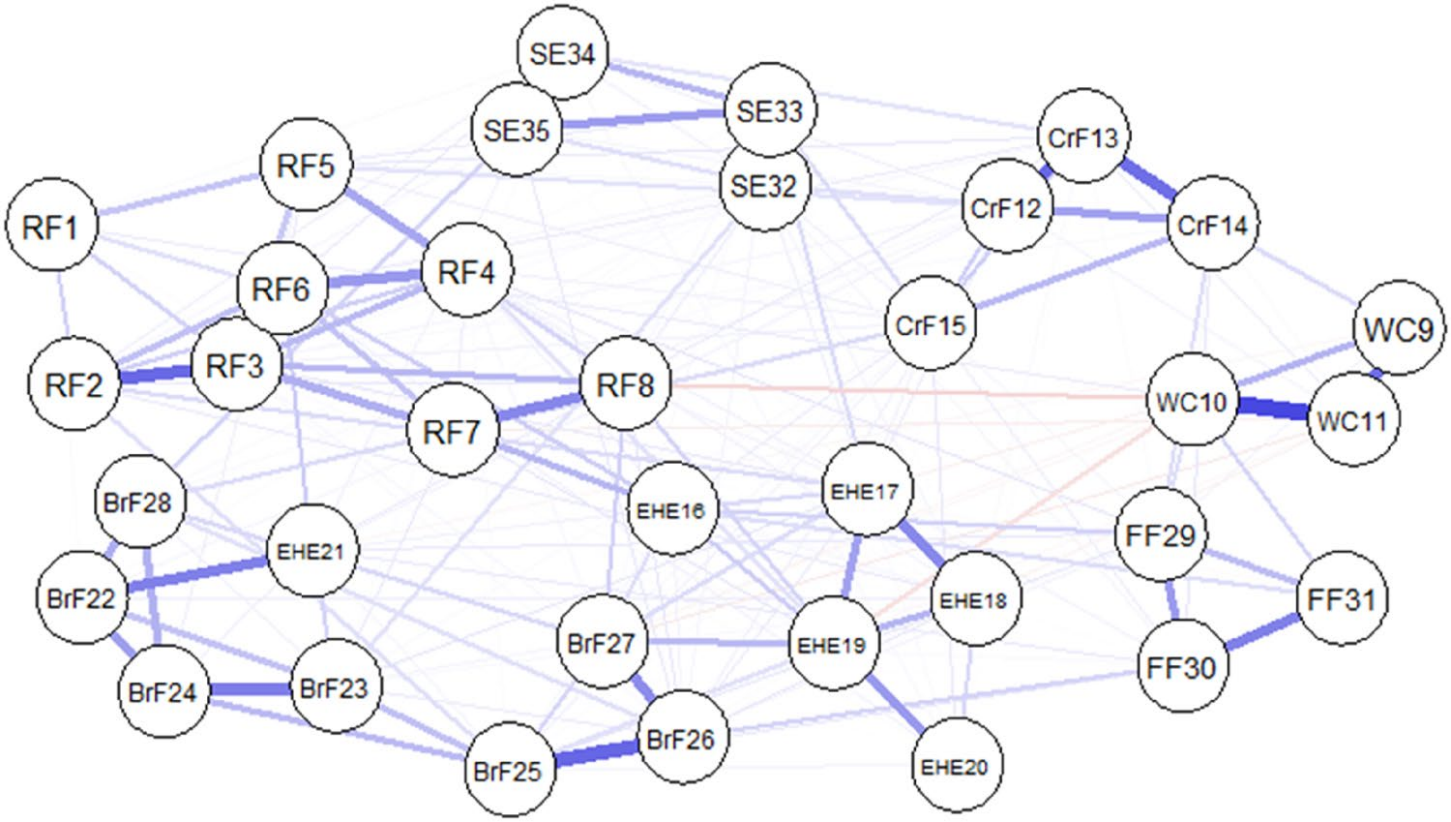
Network plot of the CPCFBS*.* The network comprises 35 items which are presented by nodes, and the edges among nodes represent their associations. The edges in blue and red are positive and negative edges, respectively, where the thick edges represent strong regularized partial correlations. CPCFBS: Chinese Preschoolers’ Caregivers’ Feeding Behavior Scale

Figure 1 shows the graphical LASSO network representing the regularized partial correlations among the 35 items, with 223 of 595 edges being non-zero. Most edges were positive correlations which were colored in blue. The stronger weights between the nodes were SE32 and SE33 (*r* = 0.55), SE34 and SE35 (*r* = 0.46), and WC10 and WC11 (*r* = 0.40). Several negative correlations were colored in red, such as RF8 and WC10 (*r* = -0.09), WC10 and EHE19 (*r* =  – 0.06), and WC11 and BrF27 (*r* =  – 0.05). We suggested that the negative edge weights were relatively small and did not indicate a corresponding association between these items.

### Community detection

**Fig. 2:**
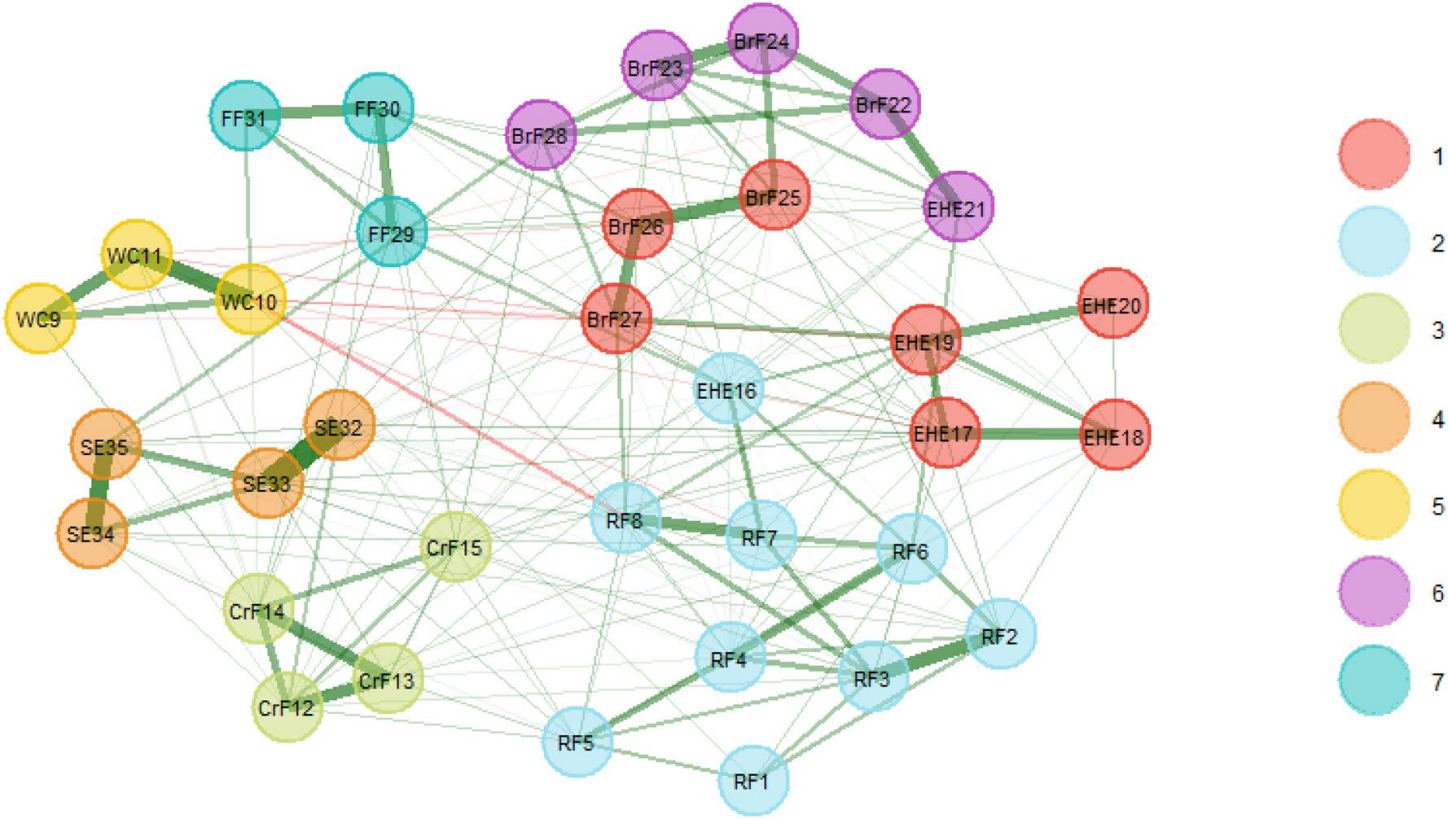
EGA Dimensionality Structure of the CPCFBS*.* The original CPCFBS was explored by EGA with 35 items in seven dimensions which were indicated by seven colors on the right side of the table. EGA: exploratory graph analysis

The EGA detected a seven-dimensional structure named EGA (all) structure; see Fig. 2. However, the dimensional attribution of items was different. EHE16 from *Encourage healthy eating* subscale clustered into Dimension2. EHE17, EHE18, EHE19, EHE20, BrF25, BrF26, BrF27 clustered into Dimension1. EHE21, BrF22, BrF23, BrF24, BrF28 clustered into Dimension 6. The items of *Behavior-restricted feeding* dimension and *Encourage healthy eating* dimension were re-clustered. The remaining items aggregated into the same dimension as the original CPCFBS; see Table 1.

**Table 1:** The original and network structure (35 items) of Chinese Preschoolers’ Caregivers’ Feeding Behavior Scale (CPCFBS)

The CPCFBS Items	Item label	Dimension	
		Original	EGA (all)
1. I will clearly tell my child what to eat and what not to eat and explain	RF1	RF	2
2. I will encourage my child to eat different kinds of food and tell her or him the nutritional value of different foods and the benefits for her or his growth	RF2	RF	2
3. I will focus on nutritional value when buying food	RF3	RF	2
4. I will try to include a variety of foods when preparing three meals a day	RF4	RF	2
5. I will give my child three meals per day regularly	RF5	RF	2
6. I will avoid buying sweets or desserts that I don’t want my child to eat	RF6	RF	2
7. I am responsible for deciding what my child eats	RF7	RF	2
8. I ask my child to have breakfast on time every day	RF8	RF	2
9. I am concerned about my child becoming overweight	WC9	WC	5
10. I am concerned about my child dieting to maintain a desirable weight	WC10	WC	5
11. I am concerned about my child eating too much when I am not around her or him	WC11	WC	5
12. I have to be sure that my child does not eat too many high-fat foods	CrF12	CrF	3
13. I have to be sure that my child does not eat too many of her or his favorite foods	CrF13	CrF	3
14. I have to be sure that my child does not eat too many sweets (candy, ice cream, cake or pastries)	CrF14	CrF	3
15. I intentionally keep some junk foods out of my child’s reach	CrF15	CrF	3
16. I will serve my child fresh vegetables and fruits each day	EHE16	EHE	2
17. I will serve my child fish products each day	EHE17	EHE	1
18. I will encourage my child to try new foods	EHE18	EHE	1
19. I will encourage my child to eat healthy foods	EHE19	EHE	1
20. I will try to ensure that each meal has a fixed time and place	EHE20	EHE	1
21. I will try to ensure that my child eat healthier, I will prepare each meal carefully	EHE21	EHE	6
22. When having a meal with my child, even if I am not hungry, I will eat on time	BrF22	BrF	6
23. When having a meal with my child, I won’t play with my mobile phone or watch TV	BrF23	BrF	6
24. When having a meal with my child, I won’t have leftovers	BrF24	BrF	6
25. When having a meal with my child, I won’t eat for more than 30 min per meal	BrF25	BrF	1
26. When having a meal with my child, I will restrict myself from eating or drinking too much at one meal	BrF26	BrF	1
27. When having a meal with my child, I usually avoid eating snacks and sweets that I don’t want her or him to eat	BrF27	BrF	1
28. When having a meal with my child, even if I don’t like them, I will try to show my willingness to eat a variety of healthy foods	BrF28	BrF	6
29. At mealtime, I will try to get her or him to eat all of the food on her or his plate in some way (e.g., persuading, playing, or praising)	FF29	FF	7
30. At mealtime, I will try to have her or him eat even if my child says she or he is not hungry	FF30	FF	7
31. I have to be especially careful to make sure my child eats enough	FF31	FF	7
32. I will supervise my child so she or he drinks less (e.g., cola, pulpy juices)	SE32	SE	4
33. I will supervise my child so she or he eats less snack food (e.g., potato chips, cheese puffs)	SE33	SE	4
34. I will supervise my child so she or he eats less high-fat food (e.g., beef jerky, sausage, fried food)	SE34	SE	4
35. I will supervise my child so she or he eats less sweet food (e.g., candy, ice cream cake, pies, pastries)	SE35	SE	4

Note. Original: the original CPCFBS structure. EGA (all): the network structure with all CPCFBS items identified by exploratory graph analysis. Subscale description: responsibility for feeding = RF = 2; weight concerns = WC = 5; content-restricted feeding = CrF = 3; behavior-restricted feeding = BrF = 6; encourage healthy eating = EHE = 1; forced feeding = FF = 7; supervise eating = SE = 4

Network loading for items on each of their dimensions was in the moderate and large range, with only EHE16, EHE20, RF1 obtained a value of less than 0.15 (small) in their primary dimensions; see Table 2. In addition, EHE16 displayed substantially equivalent cross-loadings in Dimension 1, 2, 7, which were small network loadings to these dimensions.

**Table 2:** Dimensions identified by exploratory graph analysis and network loadings for each item

Item	Dimension	Network loadings						
		1	2	3	4	5	6	7
EHE19	1	**0.33**	0.11	0.00	0.00	– 0.04	0.00	0.00
BrF26	1	**0.28**	0.03	0.02	0.02	– 0.02	0.05	0.08
EHE17	1	**0.24**	0.09	0.04	0.07	– 0.02	0.03	0.02
BrF27	1	**0.22**	0.07	0.00	0.01	– 0.05	0.06	0.01
EHE18	1	**0.22**	0.03	0.00	0.01	0.00	0.02	0.00
BrF25	1	**0.20**	0.03	00.02	0.00	0.00	0.16	0.07
EHE20	1	**0.13**	0.00	0.03	0.00	0.00	0.00	0.00
RF3	2	0.03	**0.38**	0.03	0.00	0.00	0.00	0.00
RF7	2	0.02	**0.27**	0.00	0.01	– 0.02	0.02	0.00
RF4	2	0.04	**0.27**	0.02	0.00	0.00	0.00	0.03
RF6	2	0.01	**0.27**	0.02	0.02	0.00	0.05	0.00
RF2	2	0.07	**0.27**	0.00	0.02	0.00	0.00	0.00
RF8	2	0.11	**0.21**	0.00	0.03	– 0.06	0.00	0.00
RF5	2	0.00	**0.19**	0.07	0.04	0.00	0.01	0.00
RF1	2	0.00	**0.14**	0.00	0.00	0.02	0.00	0.00
EHE16	2	0.11	**0.10**	0.01	0.00	0.00	0.01	0.11
CrF13	3	0.00	0.04	**0.40**	0.03	0.03	0.02	0.00
CrF14	3	0.00	0.00	**0.36**	0.01	0.06	0.00	0.08
CrF12	3	0.02	0.03	**0.34**	0.07	0.00	0.03	0.00
CrF15	3	0.05	0.05	**0.19**	0.05	0.00	0.04	0.04
SE33	4	0.04	0.04	0.04	**0.47**	0.00	0.00	0.00
SE35	4	0.02	0.01	0.01	**0.38**	0.00	0.05	0.00
SE34	4	0.00	0.03	0.04	**0.34**	0.00	0.01	0.00
SE32	4	0.03	0.02	0.06	**0.33**	0.00	0.04	0.02
WC11	5	– 0.03	– 0.01	0.03	0.00	**0.48**	0.00	0.00
WC10	5	– 0.05	0.05	0.01	0.00	**0.38**	0.00	0.11
WC9	5	– 0.01	0.00	0.04	0.00	**0.32**	0.00	0.01
BrF22	6	0.01	0.00	0.01	0.00	– 0.01	**0.40**	0.00
BrF24	6	0.07	0.01	0.00	0.00	0.00	**0.35**	0.00
BrF23	6	0.10	0.01	0.00	0.03	0.00	**0.27**	0.00
EHE21	6	0.01	0.05	0.03	0.01	0.00	**0.21**	0.05
BrF28	6	0.06	0.00	0.05	0.05	0.00	**0.17**	0.00
FF30	7	0.08	0.00	0.05	0.02	0.00	0.02	**0.34**
FF31	7	0.00	0.03	0.00	0.00	0.06	0.00	**0.29**
FF29	7	0.03	0.05	0.05	0.00	0.05	0.02	**0.25**

*Note.* The numbers in bold represent the network loadings of the main attribution dimension of items. The meaning of the abbreviations of the items refers to Table 1

### Structural consistency

**Fig. 3:**
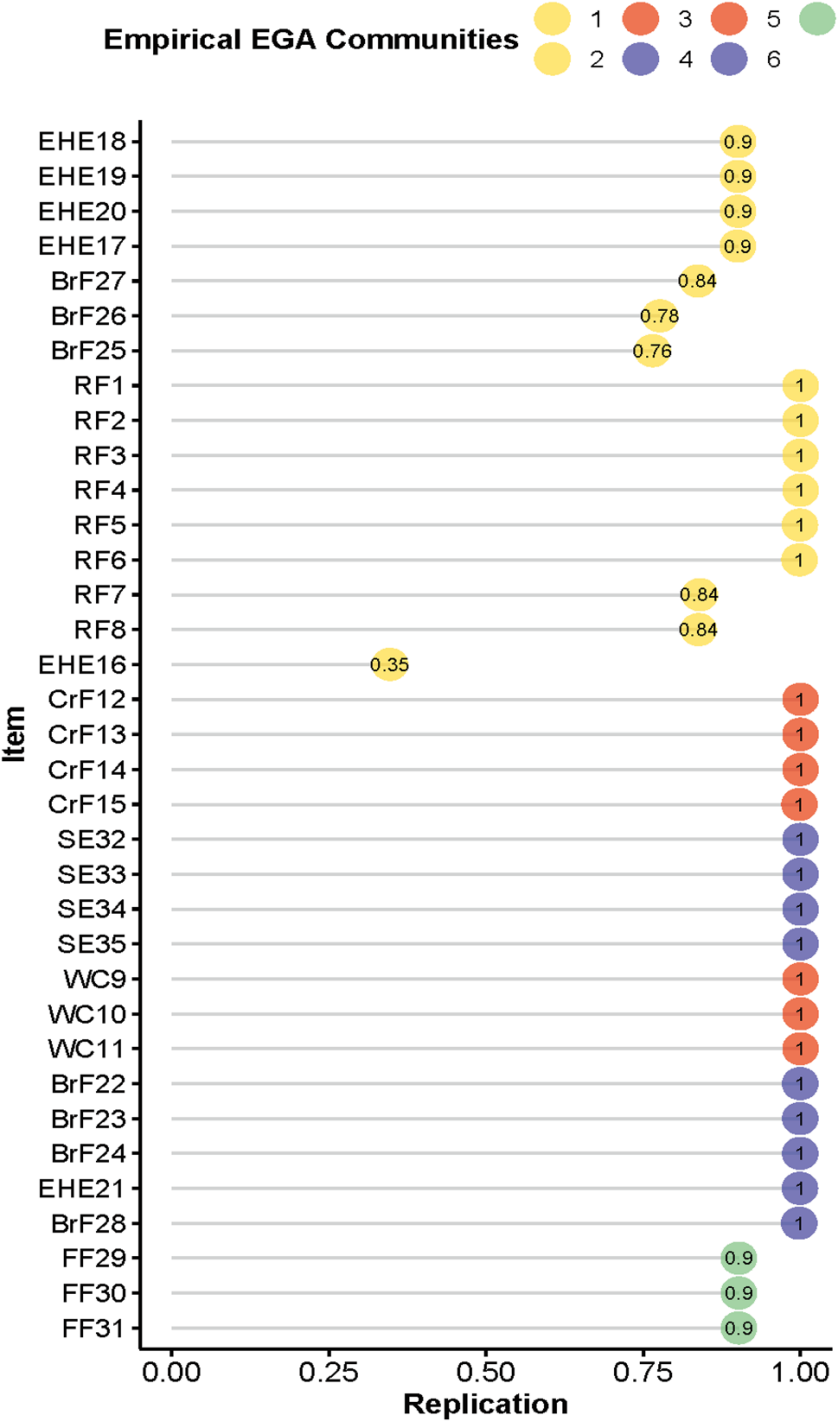
Item Stability of the EGA (all) structure. The value of 0.75 as a standard of acceptance for the dimension replication. EGA (all) structure: network structure with all CPCFBS items detected by EGA

**Fig. 4:**
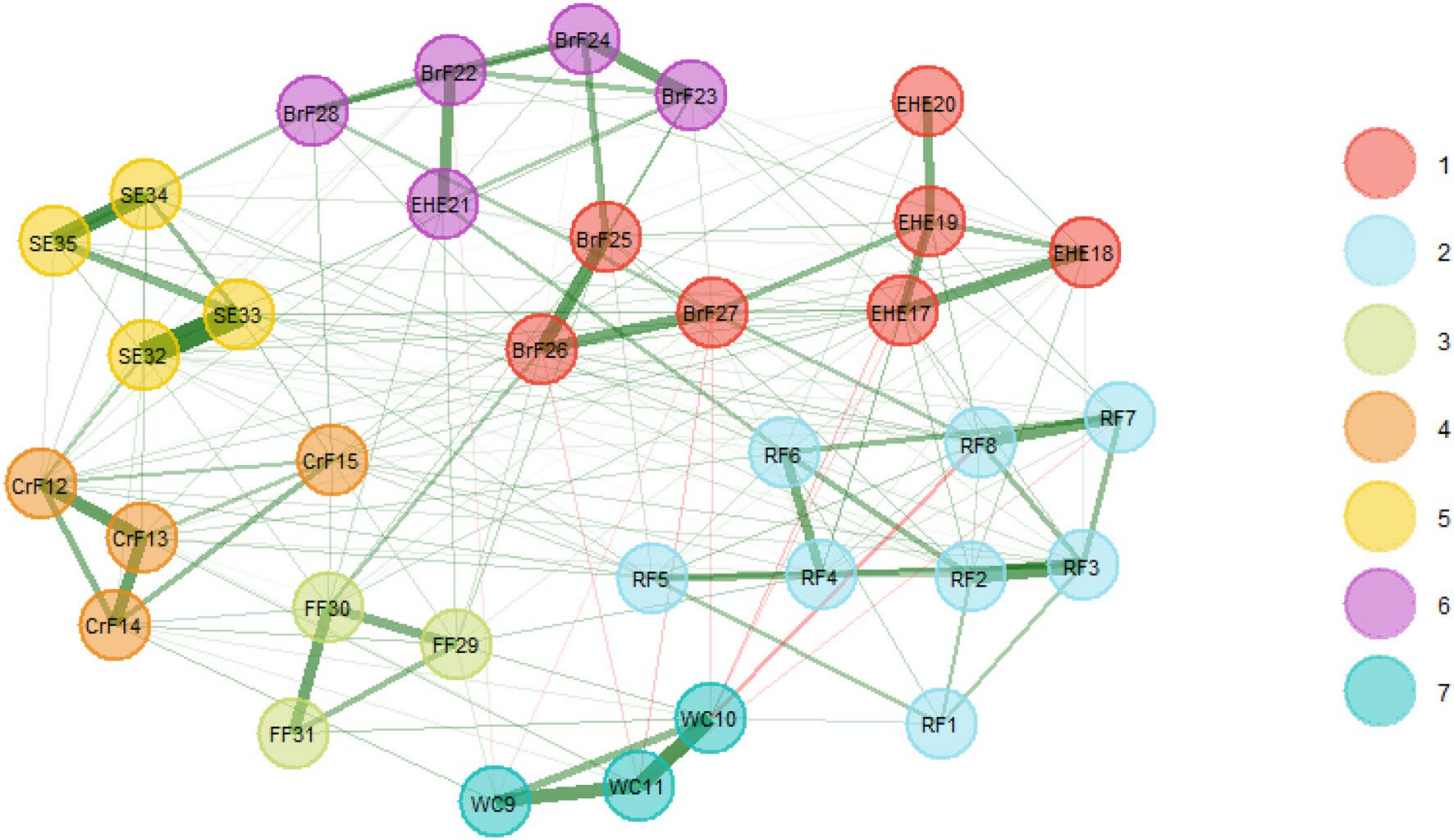
EGA (del) dimensionality structure of the CPCFBS*.* After deleting EHE 16, we detected a seven-dimensional structure composed of 34 items. EGA (del) structure: network structure detected by EGA after deleting an unstable CPCFBS item (EHE 16)

**Fig. 5:**
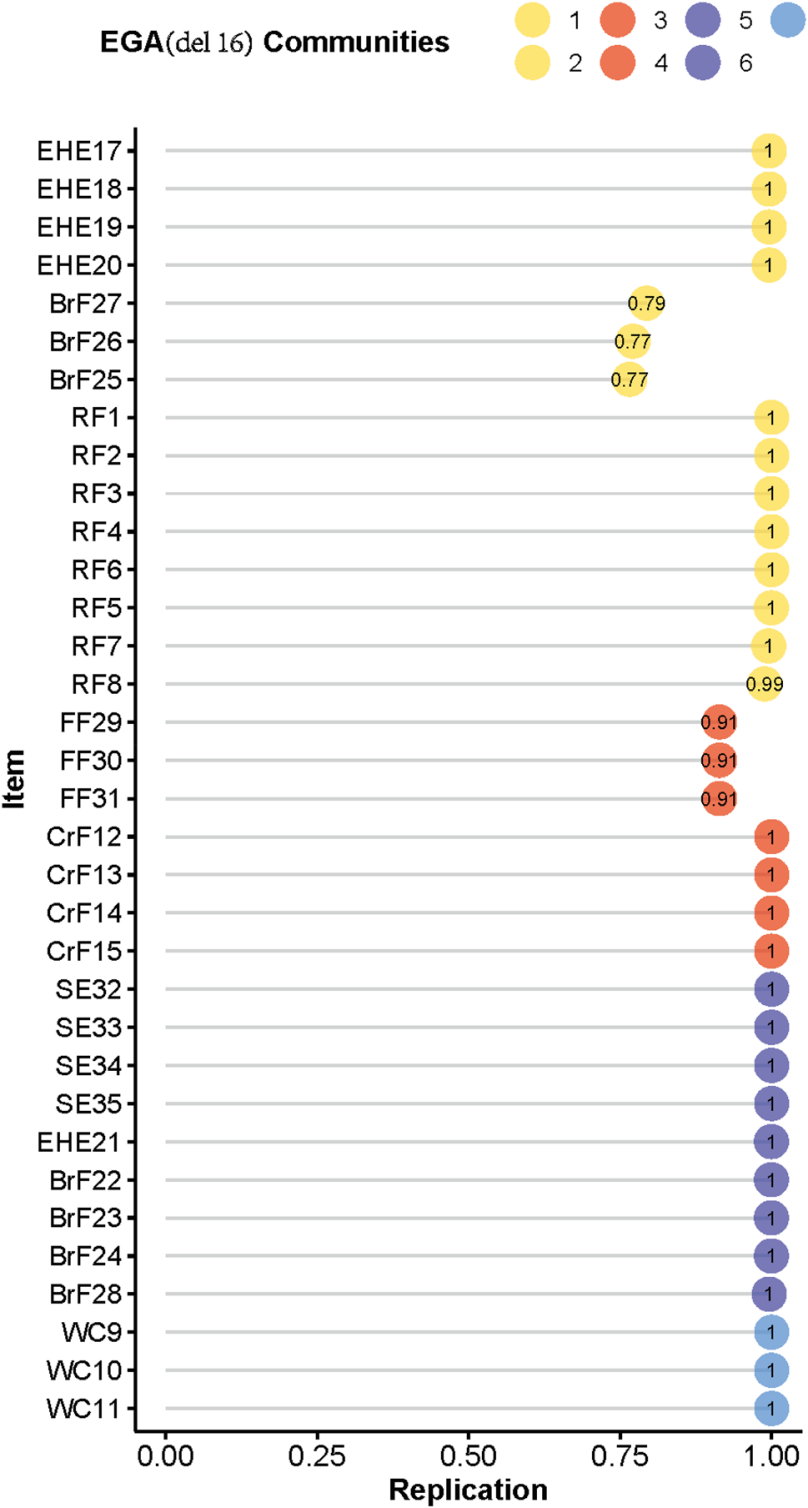
Item stability of the EGA (del) structure*.* Deleted EHE16 and re-analyzed the item stability using EGAboot

As shown in Table 3, the frequency of seven dimensions in EGA (all) structure was 0.766. Other network structures were also identified, especially the structure with six dimensions (0.112) and eight dimensions (0.114). The relatively high frequency of the six dimensions and eight dimensions illustrated that EGA (all) structure was unstable. Table 4 shows that Dimension 1 and 2 from EGA (all) structure presented low structural consistency, with value of 0.69 and 0.35, respectively. Therefore, we examined the stability of items within each dimension using the item replication; see Fig. 3. EHE16 showed relatively low item stability, with the value of 0.35. Combining with Table 2 for verification, EHE16 had low network loading in all dimensions, while item stability was also poor. This suggested that EHE16 cannot be assigned to any of the dimensions. The unstable item directly contributed to the unstable of structural consistency. To increase the consistency of the network structure, we removed EHE16 and re-analyzed the data using the same method. After removing the unstable item, we detected a final seven-dimensional structure (EGA (del) structure) composed of 34 items; see Fig. 4. The structural consistency of the EGA (del) structure was significantly improved. The frequency of the seven dimensions improved drastically from 0.766 to 0.855; see Table 3. All items replicated in their particular dimensions have a frequency of at least 0.77; see Fig. 5.

**Table 3:** Frequency of the number of dimensions identified

The number of dimensions identified	Frequency	
	EGA (all)	EGA (del)
Five	0.005	0.001
Six	0.112	0.088
Seven	0.766	0.855
Eight	0.114	0.056
Nine	0.004	0.000

*Note.* EGA (all): network structure with all CPCFBS items detected by exploratory graph analysis. EGA (del): network structure detected by exploratory graph analysis after deleting an unstable CPCFBS item (EHE 16)

**Table 4:** Structural consistency of dimensions

Dimension	Structural consistency	
	EGA (all)	EGA (del)
1	0.69	0.77
2	0.35	0.99
3	1.00	0.91
4	1.00	1.00
5	1.00	1.00
6	1.00	1.00
7	0.90	1.00

*Note.* A value of 0.75 was set as an acceptable standard for structural consistency. EGA (all): network structure with all CPCFBS items detected by exploratory graph analysis. EGA (del): network structure detected by exploratory graph analysis after deleting an unstable CPCFBS item (EHE 16)

In the EGA (del) network structure, the first dimension *Encourage healthy eating* contained seven items (EHE17, EHE18, EHE19, EHE20, BrF25, BrF26, BrF27). The second dimension *Responsibility for feeding* contained eight items (RF1, RF2, RF3, RF4, RF5, RF6, RF7, RF8). The third dimension *Forced feeding* contained three items (FF29, FF30, FF31). The fourth dimension *Content-restricted feeding* contained four items (CrF12, CrF13, CrF14, CrF15). The fifth dimension *Supervise eating* contained four items (SE32, SE33, SE34, SE35). The sixth dimension *Behavior-restricted feeding* contained five items (EHE21, BrF22, BrF23, BrF24, BrF28). The seventh dimension *Weight concerns* contained three items (WC9, WC10, WC11).

### Model fit

The model fit measures of latent variable model (the original CPCFBS structure), and the seven dimensions detected by EGA (all) and EGA (del) are displayed in Table 5. The fit indices indicated that the absolute fit and relative fit of EGA (del) structure (χ^2^ = 1777.173, *p* < 0.01, RMSEA = 0.057,CFI = 0.883, AIC = 65,500.993, BIC = 65,914.290) were better than the original CPCFBS structure (χ^2^ = 2075.635, *p* < 0.01, RMSEA = 0.061,CFI = 0.863, AIC = 67,634.554, BIC = 68,057.139) and the EGA (all) structure; meanwhile, the RMSEA of EGA (del) network model reached good benchmark according to the criteria mentioned above.

**Table 5:** Model fit comparison of the original, EGA (all) and EGA (del) structure of Chinese Preschoolers’ Caregivers’ Feeding Behavior Scale (CPCFBS)

Structure	χ^2^	*df*	*p*	RMSEA (CI_90_)	CFI	AIC	BIC
Original	2075.635	539	< 0 .01	0.061(0.058–0.064)	0.863	67,634.554	68,057.139
EGA (all)	1956.243	539	< 0.01	0.059(0.056–0.061)	0.873	67,515.161	67,937.746
EGA (del)	1777.173	506	< 0.01	0.057 (0.054–0.060)	0.883	65,500.993	65,914.290

*Note. df*: degrees of freedom; RMSEA: root-mean-square error of approximation; CFI: comparative fitness index; AIC: Akaike Information Criterion; BIC: Bayesian Information Criterion. The values of RMSEA ≤ 0.05 and CFI ≥ 0.97 indicate a good absolute fit, RMSEA ≤ 0.07 indicate an acceptable absolute fit, and CFI between 0.95 and 0.97 is considered acceptable. The lower values of AIC and BIC indicate the better relative fit. Original: the original CPCFBS structure. EGA (all): network structure with all CPCFBS items detected by exploratory graph analysis. EGA (del): network structure detected by exploratory graph analysis after deleting an unstable CPCFBS item (EHE 16)

### Reliability

The reliability of all structures is displayed in Table 6. The McDonald’s Ω of the original structure, EGA (all) structure and EGA (del) structure indicated that all structures had acceptable reliability (0.74–0.90), except for the dimension of forced feeding (0.65). The results showed that the EGA structure had nearly same values of the McDonald’s Ω with the original structure. To better determine the robustness of the results, we calculated Cronbach's alpha coefficients for the three structures. The reliability of the three structures revealed that the Cronbach’s alpha coefficient was in alignment with the McDonald’s Ω levels.

**Table 6:** The McDonald’s Ω and Cronbach’s alpha reliability of three different structure

Structure	Encourage healthy eating	Supervise eating	Forced feeding	Responsibility for feeding	Behavior-restricted feeding	Content-restricted feeding	Weight concerns	Total
McDonald’s Ω (95% CIs)								
Original	0.74(0.71–0.77)	0.87(0.85–0.90)	0.65(0.59–0.70)	0.85(0.83–0.87)	0.82(0.79–0.84)	0.82(0.79–0.84)	0.76(0.73–0.79)	0.90 (0.89–0.91)
EGA (all)	0.83(0.81–0.85)	0.87(0.85–0.90)	0.65(0.60–0.07)	0.85(0.84–0.87)	0.78(0.75–0.81)	0.82(0.79–0.84)	0.76(0.73–0.79)	0.90 (0.89–0.92)
EGA (del)	0.83(0.81–0.85)	0.87(0.85–0.90)	0.65(0.60–0.07)	0.85(0.83–0.87)	0.78(0.75–0.81)	0.82(0.79–0.84)	0.76(0.73–0.79)	0.90 (0.89–0.91)
Cronbach's alpha (95% CIs)								
Original	0.74(0.71–0.77)	0.88(0.86–0.89)	0.65(0.61–0.70)	0.85(0.83–0.86)	0.82(0.80–0.84)	0.81(0.79–0.83)	0.75(0.72–0.78)	0.91 (0.90–0.92)
EGA (all)	0.83(0.81–0.85)	0.88(0.86–0.89)	0.65(0.61–0.70)	0.85(0.83–0.86)	0.78(0.75–0.80)	0.81(0.79–0.83)	0.75(0.72–0.78)	0.91 (0.90–0.92)
EGA (del)	0.83(0.81–0.85)	0.88(0.86–0.89)	0.65(0.61–0.70)	0.85(0.83–0.86)	0.78(0.75–0.80)	0.81(0.79–0.83)	0.75(0.72–0.78)	0.90 (0.89–0.91)

*Note.* The score of McDonald’s Ω and Alpha reliability above 0.7 was considered satisfactory for internal consistency. Original: the original CPCFBS structure. EGA (all): network structure with all CPCFBS items detected by exploratory graph analysis. EGA (del): network structure detected by exploratory graph analysis after deleting an unstable CPCFBS item (EHE 16)

## Discussion

The purpose of this study was to re-explore the structure of the CPCFBS using network analysis in a large sample of preschoolers’ caregivers from China. To the best of our knowledge, this is the first study applying network analysis to the study of feeding behavior. We aimed to investigate whether a network structure would better explain the CPCFBS dimensionality and its item responses.

Many researchers have adapted the ideology of some measurements in psychology to evaluate caregivers’ feeding behavior scales. With the intersection development of psychometrics, new methods like network analysis have been widely used in psychological scales and have been approved to have remarkable advances. For instance, Hudson Golino et al. [35] investigated the structure of the Children’s Concentration and Empathy Scale (CCES) using network analysis. They refined and reassessed the CCES for better interpretation. Ribeiro Santiago et al. [35] employed a multi-method approach (traditional method and network analysis) to evaluate the EQ-5D-5L. The results of their research showed excellent psychometric properties. Therefore, it was worth to try to investigate the feeding behavior scale using network analysis. The different methods brought novel and interesting information on the structure.

In this study, we demonstrated the use of GGM to investigate relationships between items and implemented EGA to detect the dimension. After testing the structural consistency, we finally acquired a seven-dimensional network structure with 34 items. The results suggested that the clustered dimensions were slightly different in the original CPCFBS structure and the EGA (all) structure. The EGA structure appeared to have better statistical power. Our study illustrated that network analysis was a fitting method to explore the feeding behavior of caregivers for preschoolers.

In terms of network estimation, its essential feature was to focus on interrelationships among observational variables (e.g., scale items), which was not presented in the traditional structural exploration [30]. We found items with strong associations by analyzing the partial correlation of the global data. For instance, the highest weight of correlation examined for SE32 *I will supervise my child so she or he drinks less (e.g., cola, pulpy juices)* and SE33 *I will supervise my child so she or he eats less high-fat food (e.g., beef jerky, sausage, fried food)* indicated that caregivers supervised their children’s drinks and high-calorie food in the meantime. The results suggested that the analysis and guidance on caregivers’ certain feeding behaviors should extend to the items with strong correlations, since they may occur simultaneously.

The dimensionality structure detected via EGA was consistent with the original CPCFBS seven-dimensional structure, while certain items were rearranged. EGA (all) structure was unstable, since its frequencies of dimensions identified by bootEGA were dispersed and the structural consistency was relatively low. One of the reasons that some dimensions were not as stable as others was because of the low stability of items. [33]. Interestingly, EHE16 *I will serve my child fresh vegetables and fruits each day* was not consistently clustered into a unique dimension (i.e., network cross-loadings) and had a serious problem with item stability. It was probably the main factor that led to the instability of the EGA network structure. We decided to remove the unstable item to obtain a reliable network structure [EGA (del) structure]. The rearrangement of *Encourage healthy eating* items (EHE21) and *Behavior-restricted feeding* items (BrF25, BrF26, BrF27) was found in this sample. It was reasonable that the assignment of these three items to respective dimensions could be interpreted from Chinese contextual background.

The computation of model fit provided a robust support for the EGA (del) seven-dimensional structure. All the coefficients suggested that the network structure was stable enough to meet the requirements. The reliability of all three structures were consider to be equivalent and adequate. Although the network analysis eliminated a small number of items from CPCFBS, the approach enabled the CPCFBS to be more optimally structured, which was believed to be more important. The structure explored through the method of network analysis also proved that the CPCFBS scale was a well-developed scale and could be recommended for use.

## Limitations and future directions

Our results indicate that network analysis is a useful tool to explore the structure of feeding behavior. Yet, there are a few limitations worth to notice when using this method. First, network analysis is the process of capturing the associations among items and it requires the analysis of the stability of items and structures. Thus, the items are the fundamental condition for analytical process. To ensure that the final structure is both stable and reliable, the researchers need to find a balancing state between item selection and classification. Second, GGM is the cross-sectional model, so that it can only analyze the correlation of the items without interpreting the causality of the items. In future research, we could investigate network time-series analysis to study the cause of feeding behavior items. Finally, due to the lack of application of network analysis in the field, more data from different measurements should be used to replicate the results with the new network structure of feeding behaviors. Further research is needed to explore the centrality, an exclusive measurement feature for network analysis, and to address practical challenges in feeding behavior. This may help researchers to identify the priority targets (e.g., highly central items) and corresponding outcomes in different types of feeding behavior.

## What is already known on this subject?

The caregivers’ feeding behavior are associated with childhood obesity. Previous studies to develop and validate measures of caregiver’s feeding behavior provide a powerful base. Nevertheless, the feeding behavior structure estimated with the novel network analysis has not been investigated.

## What this study adds?

The current study employed network analysis to investigate the CPCFBS in large sample of Chinese preschoolers’ caregivers. A seven-dimensional scale structure was obtained by deleting one unstable item and rearranging four items. The dimensional structure was more stable than that of the original CPCFBS. The evaluation indicators of the network structure were satisfactory. Our research provided evidence that children health behavior scales can be well conceptualized as a network analysis system.

## Supplementary Information

Below is the link to the electronic supplementary material.

Supplementary file1 (DOCX 18 KB)

## Data Availability

The dataset used in the study are available from the corresponding author on reasonable request.
