# Supplementary material for: Estimating and validating the structure of feeding behavior networks
Source: Eat Weight Disord. 2022 Oct 16;27(8):3521–32. doi: 10.1007/s40519-022-01489-1 (PMC9803728; doi:10.1007/s40519-022-01489-1)
Supplement: Supplementary file 1 — Supplementary file1 (DOCX 18 KB) [file 40519_2022_1489_MOESM1_ESM.docx]

**Table S1** Demographic Information of the Samples from the Chinese Preschoolers’ Caregivers’ Feeding Behavior Scale (2016-2017, in Jinan and Xi’an City)

| Baseline characteristic | Full sample | |
| --- | --- | --- |
|  | *n* | *%* |
| Region |  |  |
| Urban | 399 | 52.0 |
| Rural | 369 | 48.0 |
| Child sex |  |  |
| Male | 410 | 53.4 |
| Female | 358 | 46.6 |
| Child age (y) |  |  |
| ≥3 -＜4 | 242 | 31.5 |
| ≥4 -＜5 | 257 | 33.5 |
| ≥5 -＜7 | 269 | 35.0 |
| Child weight status |  |  |
| Normal | 586 | 76.3 |
| Overweight | 101 | 13.2 |
| Obesity | 81 | 10.5 |
| Childe-caregiver relationship |  |  |
| Parent | 585 | 76.2 |
| Grandparent and others | 183 | 23.8 |
| Caregiver age (y) |  |  |
| 20-29 | 88 | 11.5 |
| 30-39 | 478 | 62.2 |
| 40-49 | 57 | 7.4 |
| ≥50 | 145 | 18.9 |
| Caregiver weight status |  |  |
| Normal | 546 | 71.1 |
| Overweight | 187 | 24.3 |
| Obesity | 35 | 4.6 |
| Caregiver education |  |  |
| Junior high school or below | 212 | 27.6 |
| Senior high school | 195 | 25.4 |
| College or university or above | 361 | 47.0 |
| Family monthly income ($) |  |  |
| <750 | 350 | 45.6 |
| 750-1,500 | 336 | 43.8 |
| >1,500 | 82 | 10.7 |

*Note. N* = 768. The children were on average of 4.9 years old (*SD* = 1.0). Child weight status: Normal: BMI<85th percentile, Overweight: BMI>85th percentile <95th percentile, Obesity: BMI≥95th percentile. Caregiver weight status: Normal: BMI<24, Overweight: BMI>24 - <28, Obesity: BMI≥28.
